# Environmental Risk Assessment of Nanomaterials in the Light of New Obligations Under the REACH Regulation: Which Challenges Remain and How to Approach Them?

**DOI:** 10.1002/ieam.4267

**Published:** 2020-04-28

**Authors:** Kathrin Schwirn, Doris Voelker, Wiebke Galert, Joris Quik, Lars Tietjen

**Affiliations:** ^1^ German Environment Agency (UBA), Dessau Roßlau Germany; ^2^ National Institute for Public Health and the Environment (RIVM), Bilthoven the Netherlands

**Keywords:** Nanomaterials, Environmental risk assessment, REACH, Classification

## Abstract

Within the European regulation on the Registration, Evaluation, Authorisation and Restriction of Chemicals (REACH, EC No 1907/2006) specific provisions for nanomaterials were included, which have become effective on 1 January 2020. Although knowledge on the peculiarities of testing and assessing fate and effects of nanomaterials in the environment strongly increased in the last years, uncertainties about how to perform a reliable and robust environmental risk assessment for nanomaterials still remain. These uncertainties are of special relevance in a regulatory context, challenging both industry and regulators. The present paper presents current challenges in regulatory hazard and exposure assessment under REACH, as well as classification of nanomaterials, and makes proposals to address them. Still, the nanospecific considerations made here are expected to also be valid for environmental risk assessment approaches in other regulations of chemical safety. Inter alia, these proposals include a way forward to account for exposure concentrations in aquatic toxicity test systems, a discussion of how to account for availability of dissolving nanomaterials in aquatic test systems, and a pragmatic proposal to deduce effect data for soil organisms. Furthermore, it specifies how to potentially deal with nanoforms under the European regulation on Classification, Labelling and Packaging of substances and mixtures (CLP) and outlines the needs for proper exposure assessments of nanomaterials from a regulatory perspective. *Integr Environ Assess Manag* 2020;16:706–717. © 2020 The Authors. *Integrated Environmental Assessment and Management* published by Wiley Periodicals LLC on behalf of Society of Environmental Toxicology & Chemistry (SETAC)

## KEY POINTS

- Thanks to experiences gained from intensive studies on fate and effects of nanomaterials in the environment, peculiarities of testing and assessing are known and can be considered in corresponding strategies.
- Uncertainties in environmental risk assessment remain as standard test methods for hazard assessment, revised data requirements, and adapted default values as well as monitoring data are in many cases still missing.
- While update of existing guidance to account for the specifics of nanomaterials is ongoing, however mostly not yet available, in the meantime pragmatic decision are needed to account for the uncertainties and allow a valid hazard assessment for regulatory purposes.
- The paper highlights the need for improved hazard and exposure assessment, proposes appropriate testing and analysis regime to derive more valid effect concentrations as well as demands revisions of current release estimation and advances in fate modeling and related input parameters.

## INTRODUCTION

According to the Annex I of the European Union (EU) regulation on the Registration, Evaluation, Authorisation and Restriction of Chemicals (REACH; EC No 1907/2006) (EC 2006), an environmental chemical hazard assessment (section 3) and an exposure assessment (section 5) need to be done for risk characterization of a registered substance. The aim of the environmental hazard assessment is to enable the determination of classification and labeling of a substance according to Regulation (EC) No 1272/2008 (EC 2008) as well as to predict a no‐effect concentration (PNEC) of a substance for an environmental risk assessment. For the latter, different environmental compartments and bioaccumulation have to be taken into account. For an appropriate assessment of nanomaterials, various nanospecific amendments to the REACH Annexes were implemented in 2018 (EC 2018), to be applied beginning 1 January 2020. To support registration of nanomaterials, the European Chemicals Agency (ECHA) provided nanospecific annexes to its guidance on information requirements and chemical safety assessment under REACH (ECHA 2019). Besides that, there are activities underway at the level of the Organisation for Economic Co‐operation and Development (OECD) to provide nanospecific test guidelines and guidance that will improve reliability of data for an assessment of nanomaterials.

Although numerous publications on ecotoxicity of nanomaterials have been published during the last decade (Kahru and Dubourguier 2010; Handy et al. 2012; Skjolding et al. 2016; Lead et al. 2018), robust PNECs for nanomaterials are still not available to a great extent. The main reasons are that, for example, available data based on regulatory relevant endpoints focus mainly on acute ecotoxicity whereas long‐term data are often still limited. Also, information on actual exposure during the test is often lacking, and thus, quality‐assured data on ecotoxicity for nanomaterials remain scarce. Those challenges in reliable aquatic toxicity testing of nanomaterials have already been identified, and solutions by, for example, developing test and assessment strategies were proposed (Hund‐Rinke et al. 2015; Potthoff et al. 2015; Kennedy et al. 2017). In addition, there are often ambiguities about the similarities of diverse nanoforms of a substance (those forms of a substance that fall under the European definition of nanomaterials [EC 2011]) and about whether available data can be used for a joint PNEC derivation. Still, due to the increased experiences from research, improved knowledge on test performance is available (OECD 2012; ECHA 2017a) and information requirements can be formally fulfilled.

Exposure estimation is still challenging for nanomaterials. In particular, fate and behavior processes of nanomaterials differ considerably in comparison to that of soluble (organic) substances. Experts have started to develop various models or tools for allowing realistic exposure estimation for nanomaterials. In that context, several research projects such as the EU Framework Programme (FP) 7 SUN, EU FP 7 NanoFATE, Horizon 2020 NanoFaSe, or EU LIFE NanoMONITOR (NanoSafety Cluster 2020) were concerned with exposure assessment and the applicability of release factors during life cycle steps of nanomaterials.

Even though scientific efforts of the last decade provide valuable information on the specific fate, behavior, and effects of nanomaterials in the environment, gaps remain regarding a reliable derivation of PNEC and predicted environmental concentration (PEC) as parts of environmental risk assessment. These gaps also apply to performing a reliable hazard classification of nanomaterials. The aim of the present publication is to point out these challenges and to promote discussion for a way forward for regulatory purposes, focusing mainly on the requirements for risk assessment according to the REACH regulation. Still, the nanospecific considerations made here are expected to also be valid for environmental risk assessment approaches in other regulations of chemical safety, for example, those falling within the European Food Safety Authority's (EFSA) remit. The intention of the present publication is to highlight major challenges when assessing environmental hazard and risk of nanomaterials based on existing data and to propose ways forward from a regulatory perspective. This analysis aims to promote discussions on the demands of regulatory risk assessment of nanomaterials with respect to improving data quality and at the same time dealing with uncertainty.

## CONSIDERATION OF DERIVING ADEQUATE EFFECT VALUES IN AQUATIC TEST SYSTEMS

Central challenges of environmental hazard assessment of nanomaterials are their fate and behavior processes (Kühnel and Nickel 2014; ProSafe 2017). These processes differ considerably from those of soluble (organic) substances and lead to specific types of exposure to test organisms. The state of a nanomaterial (e.g., highly dispersed, agglomerated, [partly] dissolved), degree of exposure, uptake, and bioavailability toward environmental organisms strongly depend on possible dissolution, agglomeration, and density‐driven sedimentation in the test system but also on transformation or its interaction with organisms due to its particulate nature (Hjorth et al. 2017). These aspects should be considered when deriving PNECs from toxicity test data. Based on these findings, we propose the following approaches for the aquatic ecotoxicity testing of nanomaterials.

### Nominal concentration versus measured concentration

**Figure 1:**
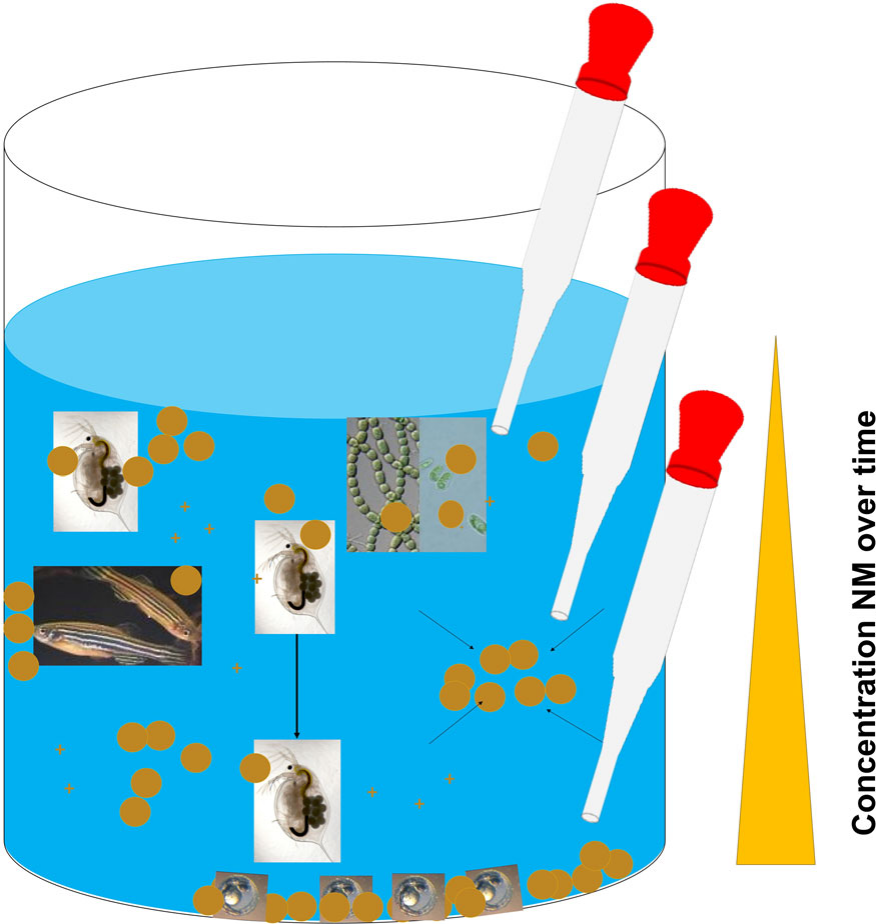
Schematic and not‐to‐scale illustration of nanomaterial (NM) behavior in tests system regarding dissolution, agglomeration, and sedimentation, which can lead to a concentration gradient over time and location and subsequently to different analytic results depending on the point (in time and location) of measurement. The presence of various test organisms in the scheme is made only for illustrative reasons to highlight the different routes of possible interactions in a space‐saving manner (circles outline the nanomaterial and (+) possible ions dissolved from the nanomaterial, the arrows illustrate the tendency of nanomaterials to agglomerate, the 3 pipettes illustrate different hypothetical sampling points along with different potential concentration situations, and the triangle beside the test system represents a concentration gradient that can evolve in the test system over time) (photos courtesy of Section IV 2.4, German Environment Agency).

In general, PNEC derivation is based on effect values using either nominal or measured concentrations. For nanomaterials, it is still unanswered which of the 2 concentration values the PNEC derivation or a classification decision, respectively, should be based on. Nominal concentrations indicate the total amount of a substance applied to the test system. However, they do not consider the change in the water phase due to the abovementioned fate processes. Thus, it is conceivable that a nominal concentration can lead to an underestimation of hazard in an aquatic system in the case in which the available amount of nanomaterials is considerably lower than the nominal concentration. Inversely, it should be noted that possibly higher local exposure due to attachment or dietary consuming of agglomerates might be overlooked when measuring the concentration of the water phase (for illustration, see Figure 1). This could potentially lead to an overestimation of environmental hazard.

We propose that for those nanomaterials that do not undergo considerable changes during the aquatic toxicity testing by agglomeration and sedimentation (i.e., dispersion stability ≥80%), derivation of hazard values based on nominal concentration should be sufficient. This proposal would follow the logic of classical ecotoxicity testing. Based on this logic, calculation of effect values based on nominal concentrations is accepted in the case in which recovery rates in the water column yield at least 80% of the nominal concentrations. For nanomaterials featuring significant differences between measured and nominal concentrations, other approaches should be taken into account: Steadiness of exposure concentration should be improved as far as feasible by, for example, flow‐through systems or frequent water renewals. Still, disadvantages of the methods should be taken into account, for example, loss of nanomaterials in tubes or valves or increase of total nanomaterial concentration in the test system over time due to sedimentation. In any case, we propose to monitor the measured concentration during the test performance at the start, at the end, and in appropriate intervals in between to derive mean values based on averaging, time‐weighted averaging, or geometric mean approaches. Further guidance on test performance and evaluation can be found in the upcoming OECD Guidance Document on Aquatic and Sediment Toxicological Testing of Nanomaterials (Mar Gonzalez, OECD, Paris, France, personal communication). In the case of water renewals, we propose to measure the concentration directly before and after water exchange.

In order to improve reproducibility and comparability of the actual exposure concentrations, appropriate frequency of measurements and correct location of measurement in the test system should be applied. In preparation for the performance of the ecotoxicity test, pretesting on, for example, dissolution and dispersion stability in the respective test media can provide supporting information on the nanomaterial's fate and behavior in the test system. Thereby, an understanding can be gained about appropriate time points and location of concentration measurement. However, for some organisms, for example, those that move throughout the entire water column and thus might face concentration gradients of the nanomaterial tested, testing at different heights at the same time points during testing might be relevant.

Still, it should be kept in mind that test organisms have an additional influence on the nanomaterial's fate. Furthermore, in the case of test media renewal, it has to be considered that the frequency of media changes could influence the average concentration of the particulate and dispersed fraction in the free water column over the whole test duration. In consequence, the longer the time period between test media renewals, the bigger the part of the investigated nanomaterial that could be dissolved or sedimented, respectively. Raw toxicity data should be carefully analyzed in relation to the obtained concentrations during testing. An analysis strategy is needed to decide on the most conclusive derivation of effect data. This strategy may include the calculation of mean concentrations (over time and location), the choice of a measured concentration appearing most suitable with regard to appearance and behavior of the test organisms, or the calculation of different effect data based on the different obtained measured concentrations and—as a conservative approach—to select the worst‐case effect data from those. In any case, a justification is needed for the chosen measured concentration to derive effect data, and the strategy for concentration measurement during testing (frequency and location) needs to be reported.

### Dissolving nanomaterials

Looking at ion‐releasing nanomaterials, it is conceivable that the contribution of the particulate or dissolved fraction to the toxicity can be misjudged. Thus, for nanomaterials that dissolve during testing, besides the measured total concentration, the concentration of the dissolved fraction also should be determined. This is of special importance in the case of inadequate media exchange frequency in relation to the dissolution rate of the investigated nanomaterial. In order to decide on appropriate media exchange frequency in relation to dissolution, pretesting on dissolution (rate) in the respective test media can provide supporting information. In avoidance of incorrect conclusions regarding the contribution of the ionic or particulate part to the observed toxicity, it is crucial to clarify in which state the nanomaterial is present during testing. A pragmatic decision on number and frequency of media renewal (time, number) is needed to increase the reproducibility and comparability of test results. Test media renewal rate based on dissolution information might be considered. This is particularly relevant in cases that intend to exclusively investigate the toxicity of the particulate fraction as well as for long‐term toxicity tests using semistatic systems.

For hazard assessment in a regulatory context, the derivation of a specific effect value (e.g., certain effect concentration [EC*x*], no observed effect concentration [NOEC]) is of central interest. The relation of the mechanism behind the found effect will be relevant only for in‐depth analysis. For particulate substances this means that, based on data collected for regulatory risk assessment use only, it usually is not differentiated between adverse effects due to the particulate state or due to the release of toxic ions. However, by neglecting the need for differentiating the actual state of the tested nanomaterial in the test system, it is neither possible to verify predictions of hazardous effects of nanomaterials based on certain properties nor to verify hypotheses on the worst‐case effect form or the “ion‐only hypothesis.” Thus, for intelligent test strategies as well as for grouping hypothesis and read‐across approaches, there is the need to distinguish physical toxicity from intrinsic toxicity, and toxicity based on the particulate nature of a nanomaterial from its toxicity based on soluble ions. Thus, depending on the rationale of hazard testing, elucidation of the contribution to toxicity needs to be considered. In that context, comparative testing with the ionic form of the investigated nanomaterials also can offer supporting information in order to examine whether ion toxicity provides a benchmark.

The aspect of dissolution of nanomaterials also plays a role in the discussion related to omitting nanospecific testing in case the nanomaterial under consideration loses its appearance due to dissolution. We propose to refrain in general from nanospecific testing if the nanomaterial under consideration completely dissolves within 12 h at the highest intended test concentration of the respective ecotoxicity test. This proposed time frame is equivalent to the half‐life time of fast‐degrading substances (OECD 2019), but in contrast it ensures that no particular fractions remain (e.g., in case the various parts of a nanomaterial's size distribution dissolve at different time scales). In such a case, the contribution to the ecotoxicological impact of the particulate form to the endpoint under investigation will very likely not be decisive. Furthermore, the question on dissolution focuses not only on the need for nanospecific testing but also on the need for long‐term data instead of acute data. In order to determine the hazard profile of poorly soluble nanomaterials as well as those not featuring a fast dissolution rate, long‐term data will be more appropriate. It is anticipated that whereas for poorly soluble substances internal concentration in the test organisms can hardly be achieved in short‐term exposure, higher internal effect levels will be possible in long‐term testing. Furthermore, the uptake and toxicokinetics of particulate substances are expected to be slower than for dissolved substances. Thus, for nanomaterials there is also the possibility that the hazard will be underestimated by acute data only. In that context it would appropriate to decide on short‐ and long‐term testing based on a default trigger value for poor solubility or slow dissolution rate, once available. However, as long as there is no scientific evidence to define test system–specific trigger values, it has to be justified case by case when to perform acute testing instead of long‐term testing. Anyway, complete and fast dissolution of a nanomaterial does not negate other possible criteria for long‐term testing.

This discussion also comes along with the question under what circumstances transformed (i.e., chemical transformation) nanomaterials should be tested rather than pristine ones. If nanomaterials transform in the test system, approaches applied for degrading substances might give guidance. Following the suggested approach, for nanomaterials with a transformation‐related half‐life time of less than 1 h, the transformation product should be tested. For these nanomaterials, testing of the pristine form might be quite challenging. Nanomaterials with a half‐life time longer than 3 d could be considered as rather stable, and the pristine form should be tested. For nanomaterials with transformation‐related half‐life times in between these boundaries, testing should be considered on a case‐by‐case basis, for example, depending on the endpoint under consideration.

### Further considerations on availability

Next to the availability of (sedimented) nanomaterials to benthic deposit‐feeders and filtering organisms (Tella et al. 2014; Kuehr et al. 2020), existing data show that agglomeration and sedimentation do not necessarily prevent availability to aquatic organisms in toxicity test systems (Botha et al. 2016; Tan et al. 2016; Hund‐Rinke et al. 2017). For instance, due to the permanently agitated test system used for testing of algae toxicity, test organisms also can be affected by unstable nanomaterials in the test dispersion. Unstable dispersed nanomaterials also can still be taken up via consuming by pelagic organisms such as *Daphnia*. However, if bioavailability and uptake of sedimented or attached nanomaterials still occur while effect values are deduced based on (the likely lower) measured nanomaterial concentrations in the water column, this can lead to worst‐case estimations of hazard and as such presents an acceptable conservative approach. Also, in the case of ion‐releasing nanomaterials, it is conceivable that released ions can get into the water phase affecting test organisms while the particulate fraction is deposited at the bottom. The understanding of bioavailability and uptake of the various nanomaterials by aquatic organisms is still far from complete and also depends, besides dissolution and agglomeration, on other aspects of the nanomaterials such as size, chemical composition, synthesis methods, or nature of coating (Lead et al. 2018). For nanomaterials, next to chemical toxicity, effects due to physical interactions also are reported, such as attachment to organisms and blocking of digestive tract or respiratory system (Jacobasch et al. 2014; Kühnel et al. 2019). On one hand, these effects can contribute to the effect values within ecotoxicity testing. On the other hand, under environmental conditions (i.e., realistic exposure concentration, availability of food) such effects may play a minor role. Still, a differentiation is nontrivial, especially under environmental settings similar to those in the test system. Because these effects are part of the very nature of nanomaterials, they should not be considered per se as artifacts of the test system. Therefore, these physical effects should be taken into account for derivation of hazard data, if they can happen under environmental conditions. Also, even under environmental conditions, it is conceivable that deposited or hetero‐agglomerated nanomaterials are fed from ground, biofilm, or plant surfaces or are taken up via sediment or natural suspended matter, respectively (Geitner et al. 2018; Perrier et al. 2018).

## CONSIDERATION OF DERIVING ADEQUATE EFFECT VALUES IN SOIL TEST SYSTEMS

Based on current knowledge of the environmental fate of nanomaterials, next to sediments, soils seem to feature one of the major sinks of nanomaterials. That makes evident the need to have a closer look at the potential hazard for soil‐living and ‐dwelling organisms. Next to the limited number of related standard test organisms (ECHA 2016b), soils feature a rather complex system that leads to major methodological difficulties when testing nanomaterials. These include the achievement of a homogenous distribution within the soil matrix to ensure uniform bioavailability to the test organisms. In addition, based on the strong interactions of the nanomaterials with the soil matrix and potentially also with the test devices, it is challenging to derive acceptable and reproducible recovery rates. Finally, due to the complexity of the soil matrix, the choice of appropriate and meaningful analytical tools is crucial, although currently still very limited. Thus, in analogy to the uncertainties in test performance for aquatic toxicity of nanomaterials, it has to be discussed and decided how to deal with these uncertainties in regulatory risk assessment.

Based on ECHA (2011) guidance in cases where soil data are not available, PNECs for soil can be derived using the equilibrium partitioning method (EPM). This method assumes similar sensitivity of aquatic and soil living organisms but considers altered availability of the substances in the soil matrix. It estimates PNECs for soil (and sediment) by employing available PNECs for water and relevant equilibrium coefficients for soil or sediment (e.g., coefficient based on organic C, *K*_oc_). Thus, it assumes homogenous distribution of the test substance in soil based on thermodynamic equilibrium, which is not the case for nanomaterials.

Therefore, alternative approaches, for example, using appropriate fate descriptors for nanomaterials in soils, are needed (Cornelis 2015). An alternative might be a coefficient describing the efficiency of particles to attach to soil matrices (which can be determined using soil column tests). It is assumed that attachment efficiency can be used as an indicator for decreased mobility and, thus, increased exposure to soil organisms. However, it still has to be elaborated whether a representative alternative coefficient for individual nanomaterials can be determined to be used in such an alternative approach, how the equation has to be designed to allow for valid derivation of PNEC soil, and how to derive PNEC soil for nanomaterials partially dissolving in soil porewater.

In order to employ EPM adapted for nanomaterials, a generally similar behavior and fate of the investigated nanomaterials in soil and water is still required. We suggest to a stepwise basis for verifying this prerequisite. This verification could be done by the analysis of material properties, which may give a first indication regarding comparable behavior in both compartments. Assuming that exposure in soils is via the water phase, the analysis could also be supported by literature review as well as pretests on dispersion stability, dissolution, and transformation in relevant aquatic media and soil porewater extracts.

However, in case there are indications for differences in behavior and fate in the aquatic and soil compartments, soil tests with all their challenges should be conducted to achieve experimental toxicity data for terrestrial organisms. In that case, it is expedient to give detailed explanations on nanomaterial preparation, test application and performance, as well as to report on the analysis strategy used and the potential limitations of the measurement methods used. In addition, recent scientific observations on soil toxicity testing highlighted increased toxicity of altered nanomaterial species in the test matrix (Diez‐Ortiz et al. 2015; Lahive et al. 2017). Related publications should be consulted in order to decide whether transformation in soil is relevant for the hazard assessment of the nanomaterial in question. In those cases, it should be considered to include comparative testing with altered nanomaterial species in the testing strategy.

## FURTHER ASPECTS OF ENVIRONMENTAL HAZARD ASSESSMENT

In general, reproducibility of data from hazard testing decreases with the complexity of the test system. Environmental hazard testing of nanomaterials tries to mimic complex environmental settings and is influenced by both the intrinsic and extrinsic properties of the investigated nanomaterial. Methodological adaptions to account for challenges in hazard testing of nanomaterials have been made. However, it is still challenging to gain reliable and reproducible data that are able to account for the potentially complex toxic action of nanomaterials. The challenge of reproducibility of test data is especially true and relevant when determining long‐term hazard as issues like stability and dissolution of nanomaterials become more relevant. Thus, and even though promising guidance for test guidelines on hazard testing is on track, the concern of misinterpretation of hazard data for nanomaterials from standardized test systems remains. We therefore propose the application of an additional safety factor of 10 as a default to account for the uncertainties of nanospecific testing in addition to the general assessment factors related to the available data basis. In cases in which the risk quotient derived from PEC/PNEC shows inacceptable risk, uncertainties and limitations of the test performance might be reviewed for a possible refinement of data. Thus, forgoing the safety factor is possible, if there is justification that test performance and quality of data are certain enough. However, based on the abovementioned challenges, such justification needs to be assessed on a case‐by‐case basis.

We assume that PNEC (and even PEC) derivation based on surface or number concentration rather than mass concentration is hardly practicable due to the analytic constraints and nonavailability of acceptable approaches to derive PEC/PNEC values from number or surface‐based concentration. However, information on toxic potential based on surface or number concentration might give room for considerations on grouping and read‐across approaches. For instance, it may help to identify gradients of toxic potential within a group of nanoforms of a substance. Reversely, the possibility of grouping would provide the advantage for a joint PNEC derivation. Grouping and read‐across approaches for nanomaterials are important issues in the field of nanomaterials safety science and regulation (ECHA 2017b; Schwirn and Völker 2019). This topic is quite complex and research is still ongoing (e.g., in projects like Gracious 2020) to understand the influence of the various properties and parameters on the toxic potential and the comparability between different nanoforms of a substance. Therefore, this issue is not discussed in detail in the present analysis.

## CLASSIFICATION ACCORDING TO CLP AND GLOBALLY HARMONIZED SYSTEM

Classification and labeling are important parts of European chemical legislation. However, the classification of nanomaterials for environmental hazards is still a challenge. Some work to handle these challenges has started under the United Nations Globally Harmonized System of Classification and Labelling of Chemicals (UN‐GHS) (UN 2014, 2018) and also in the EU (EC 2009). Besides the question of how to derive valid EC*x* or lethal concentration (LC*x*) values from the ecotoxicity tests (see *Consideration of Deriving Adequate Effect Values in Aquatic Test Systems* section), there are some additional issues that need to be considered.

The basic elements used for classification of aquatic environmental hazards are acute aquatic toxicity, chronic aquatic toxicity, potential for or actual bioaccumulation, and degradation (biotic or abiotic) of organic chemicals. We anticipate that for organic nanomaterials these general classification rules can be applied, taking into account possible specificities of particle effects (see *Consideration of Deriving Adequate Effect Values in Aquatic Test Systems* section).

For a nanoform of a substance that dissolves completely at the highest test concentration of a regarded aquatic ecotoxicity test within 12 h (see *Dissolving nanomaterials* section), we suggest that no nanospecific considerations for classification are needed, and thus, classification can be based on results of the dissolved substance. Additional concepts for poorly soluble metals and metal compounds are available in UN‐GHS (UN 2017) and in ECHA guidance (ECHA 2017c). However, these concepts focus only on the dissolved part of a substance and do not take into account possible effects of undissolved (parts of) nanomaterials.

Therefore, additional guidance is needed on how to deal with nanomaterials. We propose an adapted approach that takes into account the dissolved fraction as well as the remaining particulate fraction of nanomaterials. Given that this is in line with Annex I No 4.1.2.10.2 of the CLP regulation, no change of the legal requirements would be necessary. For non‐nanoforms, the classification of metals und metal compounds is based on the results from testing of transformation and dissolution (OECD 2001) and the known ecotoxicity of the dissolved part (metal ions) of the metal compounds. Thus, if the effect concentration of the ions is lower than the metal ion concentration from the transformation–dissolution protocol, the substance needs to be classified accordingly. In addition, metal and metal compounds are in general considered as nonrapidly degradable substances (ECHA 2017d). For nanoforms of such substances, the classification should be based on the most stringent classification of 2 approaches; these are classification based on transformation and dissolution testing of the nanoform and classification based on results from ecotoxicity testing of the nanoform. If no data are available for the nanoform, data from other comparable nanoforms or the non‐nanoform should be considered in order to be able to classify. In this case, this choice should be stated in the safety data sheet (SDS).

For the CLP classification of substances that cause long‐lasting harmful effects to aquatic life “aquatic chronic 4” (H 413), the normal approach could in principle be followed. This “safety net” classification is used when the data available do not allow classification under the formal criteria for “aquatic acute 1” (H 400) or “aquatic chronic 1” (H 410) to “aquatic chronic 3” (H 412), but there are nevertheless some grounds for concern. The CLP regulation gives an example for which substances this classification applies (annex I, table 4.1.0): poorly soluble substances for which no acute toxicity data are recorded at levels up to water solubility, and which are not rapidly degradable and have an experimentally determined bioconcentration factor (BCF) ≥500 or, if a BCF is not available, a log *K*_OW_ (coefficient on water–octanol distribution) ≥4 unless chronic toxicity NOECs > water solubility or >1 mg/L. However, nanomaterials with poor water solubility should be classified as H 413 without the need of BCF ≥500 or log *K*_OW_ ≥ 4 because BCF and *K*_OW_ are of limited relevance for nanomaterials. The potential uptake of nanomaterials into aquatic organisms currently cannot be predicted by *K*_OW_ or BCF. In the future it may be possible to consider the biomagnification factor (BMF) and dietary approaches for the classification. In the meantime, the “aquatic chronic 4” classification should be done without taking into account bioaccumulation.

For harmonized classification under the CLP regulation, further discussion is needed on how to deal with potential separate entries in annex VI of CLP‐regulation of different nanoforms. The general approach for non‐nanoforms to limit the harmonized classification under CLP to 2 entries for 1 substance may be not appropriate for nanomaterials in all cases.

## ENVIRONMENTAL EXPOSURE ASSESSMENT OF NANOMATERIALS

Beside the information on hazards, exposure estimation is essential for environmental risk assessment. Exposure estimation is based on the assessment of emission of a substance to the environment and predicting the subsequent behavior and fate. Therefore, information like the tonnage produced, application types, release rates, and pathways leading to sources of emission, as well as the knowledge on types of speciation and transformation processes are required to elucidate the extent of exposure of organisms in different environmental compartments (air, water, soil, groundwater, and sediment). This process is based on 2 types of analysis: first, the analysis of the emission of a substance to the environment and second, the fate and behavior of the substance in the environment. The challenges with both for nanomaterials are discussed in the *Emission* and *Fate* sections.

### Emission

The release of nanomaterials may occur over the entire life cycle, from manufacturing through formulation to the service life of mixtures or products, and recycling or landfilling (Gondikas et al. 2014; Kaegi et al. 2017; Giese et al. 2018). So far, only parts of the data required for exposure estimation (from release, emission, fate) are studied. For instance, there is some detailed data on nanomaterial release from products due to, for example, abrasion or weathering (Schlagenhauf et al. 2012; Canady et al. 2013; Al‐Kattan et al. 2014; Shandilya et al. 2015; Neubauer et al. 2017; Han et al. 2018). Especially from abrasion experiments of composites or coatings containing nanomaterials, it was concluded that release of free nanomaterials is unlikely. However, from an environmental perspective, the question of release concerns not only initial processes during manufacturing but also the fate of composite particles released by processes like abrasion and their potential to release free nanomaterials after entering the environment, for example, due to subsequent weathering. A further aspect of emission estimation is the impact of wastewater treatment plants to environmental emission. Various publications reported the removal efficiency of inorganic nanomaterials in model and real wastewater treatment plants, indicating that >90% and ≥70% of the amount of introduced nanomaterials were removed from the water phase, respectively, and most end up in the sewage sludge (e.g., Kaegi et al. 2013; Polesel et al. 2018; Simelane and Dlamini 2019). However, it needs to be clarified whether this is generally valid for all types of nanomaterials or whether there are differences based on chemistry or complexity (e.g., functional groups) of the nanomaterials. Another question is whether the cleaning performances regarding nanomaterials are the same for different types of wastewater treatment plants, for example, industrial treatment plants versus municipal treatment plants.

These types of data are generally applied in material flow analysis (MFA–type models that predict the transport of materials through technical compartments and release to the different environmental compartments (Caballero‐Guzman and Nowack 2016). Such MFA models can be applied to specific applications up to full life cycle considerations.

The first predictions of exposure concentrations of nanomaterials were based on these types of models, not including any specific fate processes, such as agglomeration or dissolution (Gottschalk et al. 2013; Baalousha et al. 2016; Nowack 2017). Their latest iteration also includes the states of nanomaterials that are emitted, for example, pristine, transformed, or matrix embedded (Adam et al. 2018). The main uncertainty in prediction of emission rates to the environment using MFA models comes from absence of data on the distribution of material between different products (product categories) and the lack of real‐world studies on the release from products and applications and thus, the application of worst‐case assumptions (Caballero‐Guzman and Nowack 2016; Nowack 2017). In principle, MFAs are helpful to identify where nanomaterials end up in the different environmental compartments. However, there are scarce MFAs available that fully describe complete uses and material flows due to the divided responsibilities and obligations under REACH.

When considering ECHA Guidance on Information Requirements and Chemical Safety Assessment–Chapter R.16: Environmental Exposure Assessment (ECHA 2016a), the lack of studies on release of nanomaterials from processes, applications and products is a challenge and a better understanding of nanomaterial release is required in order to estimate their emission to the environment. Another challenge is, that in R.16 it is assumed that a chemical is emitted in the pristine form; however, the state of a nanomaterial may alter before release to the environment. Therefore, in that case it is necessary to know how the possible alteration will influence the release.

According to R.16 (ECHA 2016a), emission rates are expressed as fractions of the used amount of a substance released to a specific environmental compartment. Emission rates are based on measured release rates or on default release factors. The release rates are described at the local scale as daily or annual release rate, or at the regional scale as an annual release rate for the compartments air, water, and sediment as well as soil and groundwater. Release factors are commonly default values once defined in technical guidance. They are associated to the environmental release categories (ERCs), sector‐specific ERCs (spERCs), or other factors from international or sector‐specific guidance (OECD), emission scenario documents (ESDs), and technical guidance documents (TGDs). Release factors are based on the assumptions about process parameters, for example, probability, time, temperature, and type of processing. In general, to derive the release factors during the life cycle of a chemical, information about the annual tonnage, life cycle stage and associated application, the distribution of the chemical on the market, emissions by time and space, emission paths, receiving environmental compartments, and regulatory management measures to reduce emissions must be taken into account. The default release factors provided in R.16 are conservative; however, their applicability to nanomaterials needs to be evaluated. Thus, the applicability of the sectoral spERCs needs to be evaluated. The aforementioned release categories rely on substances in non‐nanoform (same with international or further sector‐specific guidance).

First suggestions for the adaption of release factors for nanomaterials were made by the LIFE NanoMONITOR project (NanoMONITOR 2019) for all release factors from the R.16 ERC list by applying transfer coefficients (TCs). The TC is the determined percentage of a nanomaterial flowing from 1 compartment to another. The project reviewed existing analytic data for several nanomaterials and derived nanospecific release factors that differ from the defaults provided in R.16. Thus, these findings highlight the need for further evaluation of the general applicability of current release factors to nanomaterials.

Additionally, the project highlighted analytical limitations for nanomaterial characteristics. The analysis for various nanomaterials in most matrices is currently limited due to either a quite high background load or interfering matrices.

Applying modeling approaches in regulatory context requires showing that they are valid and robust for their intended use. Similar to the exercise conducted as part of the LIFE NanoMONITOR project, more work on validation of the models is needed based on measured data. This may reduce uncertainties of solely estimated data and may increase the accuracy of modeled concentrations in the environment.

### Fate

Upon the release and resulting emission of nanomaterials to the environment, the exposure of organisms depends on the fate and behavior of the nanomaterials. For nanomaterials, important factors determining the environmental fate are those that convert a nanomaterial to a different state or form (Peijnenburg et al. 2015; Baun et al. 2017). Factors or processes affecting the state of a nanomaterial are, for instance, dissolution, (hetero‐)agglomeration, or chemical transformation (i.e., sulfidation) of nanomaterials. Additionally, transport due to sedimentation or deposition plays an important role for substances in a particulate form. Thereby, estimation of exposure of nanomaterials considerably differs from that of soluble (organic) substances, given that the fate and behavior of soluble substances is based mainly on the assumption of thermodynamic equilibrium partitioning and degradation. In contrast, Praetorius et al. (2012) as well as Meesters et al. (2013, 2014) showed that, for nanomaterials, rate constants describing the relevant fate processes should be used instead.

Several publications have presented fate models that predict exposure concentrations to nanomaterials (Liu and Cohen 2014; Meesters et al. 2014; Quik et al. 2015; Garner et al. 2017; Knightes et al. 2019; Williams et al. 2019). These models range in their complexity of fate processes included, spatial and temporal resolution (Sørensen et al. 2019), and thus vary in relevance for regulatory purposes (Van de Meent et al. 2014).

An overarching challenge in addition are the data requirements for each of these fate models. Starting with the relevant estimate of release to the environment, there are nanomaterial‐specific input parameters that need to become available, potentially also distinguishing nanomaterial forms (Wigger and Nowack 2019).

Calculation of exposure concentrations of nanomaterials obviously becomes more complex as the different environmental compartments in reality have very heterogeneous characteristics, which affect the relevant endpoints (dissolution, dispersion stability, transformation) that have to be considered (e.g., marine versus fresh water or high and low content of organic matter [OM]). Because these endpoints strongly depend on the media characteristics, data for only 1 medium or condition will not fully describe the range of conditions present in the natural environment. This is not an issue specific for nanomaterials; for instance, in the European Union System for the Evaluation of Substances (EUSES) (EC 2019), a regional model scenario based on SimpleBox (Van de Meent 1993) calculates a regional background PEC, and a separate scenario is used to predict a local PEC. The sum of both the background and local PECs is used in further risk assessment. But there is some lack in methods to derive the nanospecific input parameters. Currently there is no approach to easily account for such variations, similar to existing qualitative structure–activity relationships (QSARs) derived for relating *K*_OW_ and OM content to the relevant partitioning coefficient. A potential solution is the use of multiple data sets as input parameters in fate modeling. However, this may lead to a set of raw data to be further processed for decision making. Further knowledge is necessary to be able to decide whether worst‐case estimates have to be carried out with the data obtained or whether a probabilistic estimate can be made.

Until today there is no nanospecific guidance available on which approaches to take for estimating a PEC specifically for nanoscale substances within the context of REACH, other than reference to relevant fate processes in Appendix R7‐1 (ECHA 2017a). Such guidance that specifically targets nano‐intrinsic problem areas is needed to perform realistic estimation of the environmental exposure of nanomaterials. It is solely possible to derive the PEC for the nanoscale chemicals as for the non‐nanoscale substances by following the requirements of the R.16, without considering the intrinsic peculiarities of these forms and the resulting characteristics in the behavior and fate in the environment (see Table 1).

**Table 1:** Steps in environmental exposure assessment based on the methods applied as part of REACH, EUSES, and described in R.16 and the current challenge for nanomaterials

Steps in exposure assessment (based on R.16)	Method	Main challenge	Suggested solution
Release assessment^a^	(specific) Environmental release categories	Question if general approach valid, default values valid	Evaluation of default values by monitoring data can be based on MFA models (Wang and Nowack 2018), monitoring data (Gottschalk et al. 2013)
Sewage treatment plant	Fate in sewage treatment plant	Adaptation required	Use of existing SimpleTREAT (Struijs 2015), implementation based on experimental study review or monitoring data
Exposure estimation (including distribution and fate)	Models–Local	Adaptation required	Partially available based on experiences from: Praetorius et al. (2012); nanoDUFLOW (Quik et al. 2015); NanoFASE WSO (Lofts et al. 2019); LOTOS‐EUROS (Manders et al. 2019)
	Models–Regional	Adaptation required	SimpleBox4nano (Quik et al. 2019) Additional experiences from MendNano (Liu and Cohen 2014); NanoFATE (Garner et al. 2017)
	Measurements	Currently resource intensive and complex techniques	Development of standardized measurement protocols for environmental matrices

ERC = environmental release category; ESD = emission scenario documents; EUSES = European Union System for the Evaluation of Substances; MFA = material flow analysis; OECD = Organisation for Economic Co‐operation and Development; spERC = sector‐specific ERC.

^a^ For the sake of completeness it should be mentioned that in addition to ERCs and spERCs according to R.16 (ECHA 2016a), further published information such as OECD ESDs or site‐specific information can be used for release estimation. As with the ERCs and spERCs, ESDs need to be examined for their applicability to nanomaterials.

Refinements or verification of nanomaterial release estimation are possible, based on the experiences gained from models taking into account nanospecific peculiarities or on the basis of measured release rates. Until refinements are implemented for the exposure estimation of nanomaterials and the derivation of PEC under REACH, it seems that an operation with assumptions following the worst‐case defaults given in the R.16 is in general sufficiently protective.

For the modeling approaches in predicting PECs at the regional scale, SimpleBox4Nano is fed with substance‐specific information and resulting behavior for the currently relevant nanomaterials like zinc oxide, Ag, and titanium oxide (Meesters et al. 2014). However, for PECs at the local scale, the applied model algorithms as described in R.16 need adaptation based on the relevant nanomaterial fate models at that scale (Williams et al. 2019). Given that EUSES with its built‐in models for exposure estimation is the most commonly used model for exposure estimation, input possibilities and defaults should be adopted as fast as possible.

Parallel to the necessary adaption of R.16, R.13 (ECHA 2012a) and R.18 (ECHA 2012b) need to be changed accordingly. The R.13 “Guidance on information requirements and chemical safety assessment—Risk management measures and operational conditions” addresses risk management measures depending on the shape and size of a substance, and therefore this chapter delivers a good target point for nanospecific adaption. Because release estimation during recycling, landfill, and other waste treatment processes is the main emphasis of the R.18 “Guidance on information requirements and chemical safety assessment–exposure scenario building and environmental release estimation for the waste life stage,” a check and possible amendment of several default values within these chapters seems necessary for nanomaterials because this chapter also addresses the same questions as R.16 but for the end of life cycle stage.

Another option to derive exposure concentration is actually to use measurements of nanomaterials; however, due to analytical challenges, there is a lack of monitoring data. Nevertheless, promising tools for measurement and first proposals on how to establish measurement routines are available, and further development can be expected (Hildebrand et al. 2019). This type of data is also needed to prove the significance of model results, especially in the light of the currently large variability of modeling results (Nowack et al. 2015; Lead et al. 2018). This variability is partially overcome by projects on calibration and testing of environmental fate models, inter alia those combined in the EU NanoSafety Cluster (NanoSafety Cluster 2020) like NanoFASE or CaLIBRAte. Findings and recommendations from research projects like these should be evaluated for their regulatory relevance in follow‐up processes and implemented if considered appropriate.

To carry out the risk assessment, the quotient of PEC and PNEC needs to be derived. For nanomaterials under REACH, we propose to sum up the PEC of the various nanoforms in order to take into account the total exposure to the corresponding substance. If individual nanoforms show specific effects, those forms need to be considered additionally in order to clarify how their individual toxic potential affects the outcome of risk assessment.

## CONCLUSIONS

Knowledge on environmental behavior, fate, and effects of nanomaterials incredibly increased in the last decade. In consequence of experiences gained with testing nanomaterials, the peculiarities of testing and assessing nanomaterials in the environment became apparent and thus can be considered in testing and assessment strategies. Thus, environmental risk assessment of nanomaterials is possible. However, as long as standardized test methods and guidance for hazard assessment, revised data requirements, and adapted default values as well as monitoring data are missing, uncertainties in the robustness of an environmental risk assessment for nanomaterials remain.

To overcome this situation, we call for taking reasonable and pragmatic decisions to account for the abovementioned uncertainties of hazard assessment for nanomaterials. This will lead to more valid, reproducible, and comparable data for hazard assessment in a regulatory context. For an appropriate hazard assessment of nanomaterial, an appropriate testing procedure and analytic regime has to be applied in order to derive valid effect concentrations as well as to understand the toxic profile of the investigated nanomaterials for possible grouping and analogy approaches.

In order to improve exposure assessment for nanomaterials, the appropriateness of current release factors needs to be critically reviewed and revised if needed, and scientific advances in modeling exposure data need to be pursued and adapted for regulatory use.

For improved exposure assessment of nanomaterials as part of the REACH regulation, exposure estimation requires both adapted approaches for release estimation as well as adapted multimedia fate models and their input parameters. Several fate models are available; standardized methods for the required input parameters still need further development.

Classification and labeling of nanomaterials can be done on the basis of the available concepts with small revisions on guidance level.

In order to improve environmental risk assessment as well as classification and labeling, we urge the updating of existing guidance to account for behavior specific to nanomaterials in order to decrease uncertainties. This is needed in order to fully take into account the impact of nanomaterials on the environment.

Knowledge transfer of experiences from ongoing but also finalized scientific projects to regulators should be continued to promote the appropriate risk assessment of nanomaterials. Next to the provision of improved data on environmental fate and effects of nanomaterials, this transfer can help to develop protocols and harmonized methods and to improve models used for regulatory risk assessments.

## Disclaimer

The views expressed in this manuscript are those of the authors and may not necessarily be regarded as an official position of the German Environment Agency (UBA) or the National Institute for Public Health and the Environment (RIVM).

## Data Availability

All information on which the content of the manuscript is based on is available via the indicated references.
